# The Growth-Promoting Effects of *Piriformospora indica* on Banana Under Different Concentrations of Phosphorus and Potassium Treatments

**DOI:** 10.3390/plants14121878

**Published:** 2025-06-19

**Authors:** Boxiang Zhao, Ruide Li, Na Tian, Qian Li, Chunzhen Cheng, Mingyuan Wang

**Affiliations:** 1 Department of Biological Engineering and Technology, College of Chemical Engineering, Huaqiao University, Xiamen 361021, China; 2226311037@stu.hqu.edu.cn; 2 College of Horticulture, Shanxi Agricultural University, Jinzhong 030801, China; 15738165972@163.com (R.L.); liqian198812@126.com (Q.L.); 3 College of Horticulture, Fujian Agriculture and Forestry University, Fuzhou 350002, China; ak80230527@163.com

**Keywords:** carbohydrates, osmoprotectants, phosphorus, *Piriformospora indica*, potassium, secondary metabolites

## Abstract

Banana plants require substantial nutrients, and their growth is significantly hindered by nutrient deficiency. This study investigated the influences of *Piriformospora indica* colonization on the growth of clean river sand-cultivated banana plants under varying phosphorus (P) and potassium (K) levels. Banana plants without (CK) and with *P. indica* colonization (PI) were watered using Hoagland solutions with four gradients of P or K (100%, 50%, 25%, and 0%). Results showed that *P. indica* colonization promoted the growth of banana plants under different concentrations of P and K treatments. Further analysis revealed that the pseudo-stem P and K contents were very significantly (*p* < 0.01) positively and positively correlated with biomass-related parameters (aboveground part fresh weight, root fresh weight, and total plant biomass), respectively. Root starch, sucrose, proline (PRO), and anthocyanins contents, as well as leaf malondialdehyde (MDA) and PRO contents, were positively correlated with most growth-related parameters. However, root and leaf flavonoid contents, total antioxidant capacity (T-AOC), and leaf anthocyanin content showed negative correlations with growth-related parameters. Moreover, a very significant negative correlation (*p* < 0.01) was identified between root T-AOC and root starch content. Additionally, *P. indica* altered the P and K reduction-caused starch content change patterns in both leaves and roots. Our study demonstrated that *P. indica* colonization promoted the growth of banana under different concentrations of P and K treatments by mediating the accumulation of carbohydrates, secondary metabolites, osmoprotectants, and so on.

## 1. Introduction

*Piriformospora indica* (also named as *Serendipita indica*) is an arbuscular mycorrhizal fungus (AMF)-like endophytic fungus with a broad host range [1,2]. Like arbuscular mycorrhizal fungi, the plant growth-promoting and stress tolerance-enhancing effects of *P. indica* have been confirmed in many of its host plants [3,4]. It is worth noting that *P. indica* can be cultured axenically on numerous artificial media, which makes its applications much easier than the uncultivable arbuscular mycorrhizal fungi [2]. Therefore, it has been considered an ideal module fungus for analyzing the mutualistic symbiosis mechanisms and can be utilized as a successful bio-stimulant and bio-protector for plants [5].

Evidence reveals that *P. indica* can promote plant growth by enhancing host plants’ root nutrient uptake and utilization abilities [6,7,8]. Phosphorus (P) is vital for plant growth and metabolism, and its deficiency can severely hinder the growth of plant species [9]. In the *P. indica* genome, there is a high-affinity *phosphorus transporter* (*PiPT*) gene, which can improve the P nutrition ability of host plants under P-limited conditions [9]. Studies indicate that *P. indica*-colonized plants generally exhibit higher nutrient uptake abilities than noncolonized controls [10]. For example, *P. indica* colonization promotes the P uptake ability and growth of tea plants by upregulating the expression of cytokinin-, IAA-, and P transporter-related genes [11]. Under low P conditions, *P. indica* colonization can increase the P accumulation in *Brassica napus* roots and shoots [12]. Potassium (K) can activate dozens of enzymes involved in photosynthesis, biosynthesis, and translocation of carbohydrates, and plays an important role in mediating plant growth and stress tolerance [13]. Insufficient K supply greatly impacts the growth and yield of crop plants [14]. Research shows that *P. indica* can also improve the K absorption and utilization of host plants. For example, *P. indica*-colonized pineapple plants show better K uptake ability and growth than non-inoculated controls under low P conditions [15].

Banana (*Musa* spp.) has been proven to be an ideal host plant for *P. indica* [16]. Applying *P. indica* alone or together with *Azotobacter chroococcum* significantly improves the plant height, pseudo-stem width, mean leaf number and area, leaf phosphorus, nitrogen and chlorophyll contents, and fruit yield of banana plants [16]. Moreover, the fungal colonization can promote the growth of both tissue-cultured [17] and transplanted banana plants [18,19] and enhance the high temperature [20,21] and Fusarium wilt resistance [22,23] of banana. These studies indicate that *P. indica* has great potential to be applied in banana production. As a large herbaceous plant, banana requires substantial water and nutrients from the soil for growth [24]. Insufficient P or K supply reduces the total dry matter production and distribution, growth, and yield of banana plants [25]. Previous studies found that *P. indica* colonization can alleviate short-term (in four weeks) low P-, K-, and calcium-induced damages and enhance photosynthetic rates, thereby improving the nutrient-deficiency tolerance and promoting the growth of banana [26,27]. In this study, we investigated the influences of *P. indica* colonization on the growth of clean river sand-cultivated banana plants watered with Hoagland solutions containing 100%, 50%, 25%, and 0% P or K elements for a longer time (six months). After being treated with varying P or K concentrations, banana plants with (PI) and without (CK) *P. indica* colonization were subjected to determinations of growth-related and physio-biochemical parameters. Then, to identify key *P. indica*-regulated factors influencing banana growth, correlation analysis, principal component analysis (PCA), and redundancy analysis (RDA) were performed on these determined parameters. The results obtained in this study will be helpful for understanding the promoting effects of *P. indica* on the growth of banana at different P and K levels.

## 2. Results

### 2.1. Effects of P. indica on Banana Phenotypes Under Varying P and K Concentrations

**Figure 1:**
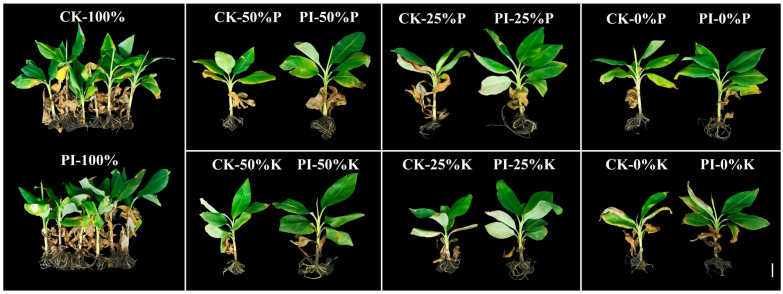
Phenotypes of *P. indica*-colonized (PI) and noncolonized (CK) banana plants at six months post-treatment with different concentrations of P or K treatments. Bar = 5 cm.

At six months post-treatment, banana plants from different groups displayed obvious phenotypic differences (Figure 1). Notably, under the same P or K concentration, the root systems of PI plants were observed to be larger than their corresponding CK plants, indicating that *P. indica* colonization promoted the root growth of banana plants at different P and K levels. As the concentrations of P or K decreased, the number of withered leaves increased, and symptoms of leaf yellowing (caused by P deficiency) or browning (caused by K deficiency) became progressively more severe in both CK and PI plants. Under the same P or K concentration, banana plants from the CK group showed more severe necrosis in old leaves compared to the PI group. Under treatments with 50%, 25%, and 0% P or K concentrations, the PI group displayed obviously better growth than the CK group. These findings indicate that *P. indica* colonization mitigates the inhibitory effects of reduced P or K concentration on banana plant growth.

### 2.2. Impact of P. indica on Banana Growth-Related Parameters Under Varying P and K Concentrations

By measuring growth-related parameters of banana plants, we further investigated the influences of *P. indica* colonization on banana growth under different concentrations of P or K treatments. For the CK plants treated with different concentrations of P (CK-P), except for root length (showed a ‘rise–fall’ pattern, peaking in the CK-25%P group) and leaf width (gradually declined as the P concentration decreased), all the other growth-related parameters showed a ‘rise–fall–rise’ change pattern, ordering in CK-100% > CK-25%P > CK-50%P > CK-0%P. For the PI plants treated with different concentrations of P (PI-P), except for pseudo-stem width (showed a ‘fall–rise–fall’ pattern, peaking in the PI-25%P group) and root length and fresh weight (declined as the P concentration decreased), all their other growth-related parameters displayed a ‘rise–fall’ trend, peaking in the PI-25%P group. Like the CK-P plants, the CK plants treated with different concentrations of K (CK-K) showed the same ‘rise–fall–rise’ change patterns in all parameters except for root fresh weight (decreased as the K concentration was reduced). For the PI plants treated with different concentrations of K (PI-K), their plant height, root length, root fresh weight, aboveground part fresh weight, and plant height decreased as the K concentration was lowered. Their leaf length, leaf width, and pseudo-stem width, however, peaked in the PI-50%K group. These findings indicated that P and K concentrations greatly influenced banana plant growth, and their influences on CK and PI plants varied a lot.

Under the same concentration of P treatment, except for the leaf length, aboveground part fresh weight, and total plant biomass at the 100% level, as well as root length at the 0%P level, the other growth-related parameters of the PI groups were all higher or significantly higher (*p* < 0.05) than those of the CK groups (Table 1). Particularly, the pseudo-stem width of the PI-100% group was significantly higher than that of the CK-100% group (about 1.11-fold). Plant height, leaf length, root number, root fresh weight, aboveground part fresh weight, and total plant biomass of the PI-50%P group were significantly higher than those of the CK-50%P group (1.55-, 1.21-, 1.34-, 2.26-, 2.07-, and 2.11-fold, respectively). Plant height, leaf length, root fresh weight, and total plant biomass of the PI-25%P group were significantly higher than those of the CK-25%P group (1.43-, 1.25-, 1.89-, and 1.84-fold, respectively). Leaf width of the PI-0%P group was greater than that of the CK-0%P group (1.43-fold).

By comparing the growth-related parameters of PI-K banana plants, we found that the plant height, leaf length, leaf width, and pseudo-stem width of PI-100%K, PI-50%K, and PI-0%K were all significantly higher than their corresponding CK groups (*p* < 0.05). The root length and aboveground part fresh weight of the PI-50%K group were significantly higher than the CK-50%K group, accounting for 1.49- and 1.70-fold of it, respectively. The leaf width and root length of the PI-25%K group were significantly higher than the CK-25%K group (1.15- and 1.33-fold, respectively). Additionally, the aboveground part fresh weight, root fresh weight, and total plant biomass of PI-0%K accounted for 1.63-, 2.33-, and 1.76-fold of CK-0%K, respectively.

Collectively, our study revealed that *P. indica* colonization can promote the growth of clean river sand-cultivated banana plants under different concentrations of P or K treatments.

### 2.3. Effects of P. indica Colonization on the P and K Contents in Banana Leaves and Pseudo-Stems Under Varying P and K Concentrations

**Figure 2:**
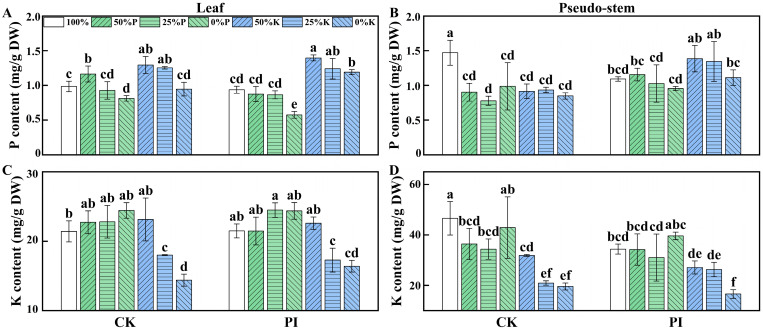
Effects of *P. indica* colonization on the P (**A**,**B**) and K (**C**,**D**) contents in leaves and pseudo-stems of banana plants under different concentrations of P or K treatments. CK: noncolonized control plants; PI: *P. indica*-colonized plants. DW: dry weight. Different lowercase letters above columns represent significant differences among samples at *p* < 0.05 level.

The P contents in leaves and pseudo-stems of banana plants from different groups were also determined (Figure 2). As the P concentration decreased, the P contents in leaves of CK plants exhibited a ‘rise–fall’ change pattern (with CK-50%P being the highest and CK-0%P being the lowest). However, the P contents in leaves of PI plants showed a gradual declining trend with the decrease in P concentration. In the CK-K and PI-K plants, the leaf P contents of CK-50%K, CK-25%K, PI-50%K, PI-25%K, and PI-0%K were significantly higher than those of CK-100% and PI-100% (*p* < 0.05). In pseudo-stems, the P content of CK-100% was higher than in all the other groups. Notably, the P contents in pseudo-stems of PI-50%P and PI-25%P groups were higher and significantly higher (*p* < 0.05) than their corresponding CK groups, respectively.

We further analyzed the K contents in banana leaves and pseudo-stems. With the decrease in the K concentration, the K contents in leaves of both CK and PI plants showed a ‘rise–fall’ change pattern (peaking at 50%P), while the K contents in pseudo-stems gradually declined. For CK-P and PI-P plants, the K content in leaves of CK-100% was lower than all other groups, while the K content in pseudo-stems of CK-100% was the highest among all groups.

### 2.4. Effects of P. indica on Starch and Sucrose Accumulations in Banana Roots and Leaves Under Varying P and K Concentrations

**Figure 3:**
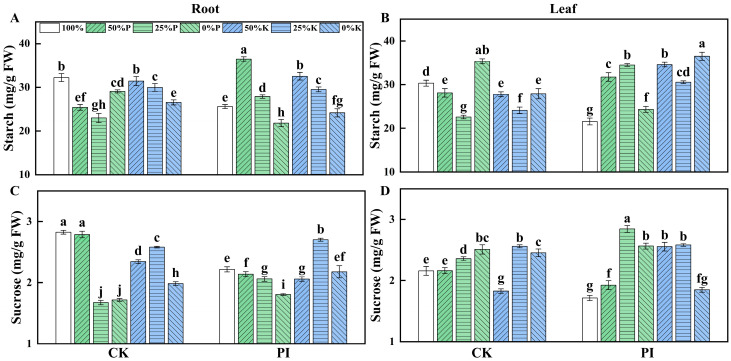
Effects of *P. indica* colonization on the starch (**A**,**B**) and sucrose (**C**,**D**) contents in roots and leaves of banana plants under different concentrations of P or K treatments. CK: noncolonized control plants; PI: *P. indica*-colonized plants. FW: fresh weight. Different lowercase letters above columns represent significant differences among samples at *p* < 0.05 level.

Nutrient deficiency significantly influences the transportation and accumulation of primary photosynthetic products in plant organs. The starch and sucrose contents in roots and leaves of CK and PI banana plants under different P or K treatments were measured (Figure 3A–D). As the P concentration decreased, CK plants showed a ‘fall–rise’ trend in root and leaf starch contents, while PI plants exhibited a ‘rise–fall’ trend. As the K concentration decreased, the leaf starch content of CK plants reduced, while the leaf starch content of PI plants showed a ‘rise–fall’ trend. For CK plants, root starch content was the highest in CK-100%, which was significantly higher than that in CK-50%P, CK-25%P, CK-0%P, PI-25%K, and PI-0%K groups (*p* < 0.05). However, the leaf starch content of CK-0%P was the highest among all the P or K-treated CK groups. For PI plants, root starch content peaked at 50%P, followed by 50%K, while the leaf starch content in PI-0%K was significantly higher than all other PI groups (*p* < 0.05).

The leaf sucrose content in the CK groups gradually increased with decreasing P concentration. In CK-25%P and CK-0%P groups, root sucrose contents were significantly lower, while their leaf sucrose contents were significantly higher than the other two CK-P groups (*p* < 0.05). For PI plants, root sucrose content decreased with decreasing P concentration and was significantly lower than that in CK-100%P and CK-50%P groups (*p* < 0.05). Leaf sucrose contents in the PI groups showed a ‘rise–fall’ trend, peaking in PI-25%P. The PI-100%P group has significantly lower leaf sucrose content than all CK and PI-P groups (*p* < 0.05). For both CK and PI plants, their root sucrose contents exhibited a ‘fall–rise–fall’ pattern with decreasing K. However, their leaf sucrose content change patterns differed: a ‘fall–rise–fall’ pattern for CK plants versus a ‘rise–fall’ pattern for PI plants.

Additionally, we found that both the starch and sucrose contents in the root and leaf of PI-100% were significantly lower than in CK-100% (*p* < 0.05). The PI-50%P group had significantly higher root and leaf starch contents but lower sucrose contents than the CK- 50%P group (*p* < 0.05). The PI-25%P group had significantly higher root and leaf starch and sucrose contents than the CK-25%P group (*p* < 0.05). The root and leaf starch contents of the PI-0%P group were significantly lower, while its sucrose contents were significantly higher than the CK-0%P group (*p* < 0.05). For K-treated plants, root starch content in PI-0%K was significantly lower than in CK-0%K. However, leaf starch contents in PI-50%K, PI-25%K, and PI-0%K were significantly higher than in their corresponding CK groups (*p* < 0.05). The root sucrose content in PI-50%K was significantly lower than in CK-50%K, while the root sucrose contents in PI-25%K and PI-0%K were significantly higher than in their corresponding CK groups (*p* < 0.05). The leaf sucrose content in PI-50%K was significantly higher than in CK-50%K, while the leaf sucrose in PI-0%K was significantly lower than in CK-0%K (*p* < 0.05).

These results demonstrated that *P. indica* colonization altered the starch and sucrose accumulations in banana roots and leaves across various P and K concentrations.

### 2.5. Effects of P. indica on Osmoprotectants Contents and Total Antioxidant Capacity in Banana Roots and Leaves Under Varying P and K Concentrations

**Figure 4:**
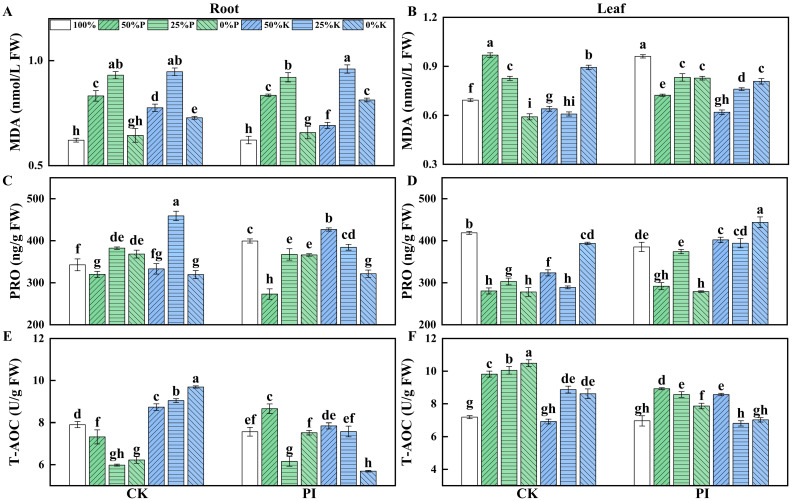
Effects of *P. indica* colonization on the MDA (**A**,**B**) and PRO (**C**,**D**) contents and T-AOC (**E**,**F**) in roots and leaves of banana plants under different concentrations of P or K treatments. CK: noncolonized control plants; PI: *P. indica*-colonized plants; FW: fresh weight; MDA: malondialdehyde; PRO: proline; T-AOC: total antioxidant capacity. Different lowercase letters above columns represent significant differences among samples at *p* < 0.05 level.

Osmoprotectants, such as malondialdehyde (MDA) and proline (PRO), play vital roles in plant stress responses. Our study revealed that, in roots of both CK and PI plants as well as in leaves of CK plants, the MDA contents displayed a ‘rise–fall’ trend with a decreasing P or K concentration (Figure 4A,B). However, the MDA contents in leaves of PI plants showed the opposite trend, peaking in the PI-100% group. Moreover, leaf MDA contents in the PI-100% and PI-0%P groups were significantly higher than in CK-100%P and CK-0%P groups (*p* < 0.05), respectively. Both the leaf and root MDA contents of PI-25%K were significantly higher than those of CK-25%K (*p* < 0.05). The root MDA content in PI-0%K was significantly higher, while its leaf MDA content was significantly lower than CK-0%K (*p* < 0.05).

The root PRO content in the PI-100% group was significantly higher than in the CK-100%P group (*p* < 0.05), whereas the root PRO content in the PI-50%P group was significantly lower than in the CK-50%P group (*p* < 0.05) (Figure 4C). The root PRO content in the CK-25%K group was the highest among all groups and was significantly higher than in all the other groups (*p* < 0.05). The root PRO content in the PI-50%K group was significantly higher than all the other PI groups (*p* < 0.05). The leaf PRO content in the CK-100% group was significantly higher than in all other CK groups (*p* < 0.05) (Figure 4D). The leaf PRO content in the PI-0%K group was the highest among all CK and PI groups. Additionally, the leaf PRO content in the PI-100% group was significantly lower than in the CK-100% group (*p* < 0.05).

The root total antioxidant capacity (T-AOC) in the CK-100% group was significantly higher (*p* < 0.05) (Figure 4E), while its leaf T-AOC was significantly lower than in the other three CK-P groups (*p* < 0.05) (Figure 4F). Both the root and leaf T-AOC in CK-K plants and the root T-AOC of PI-K plants increased as the K concentration decreased. The root T-AOC in the CK-50%K, CK- 25%K, and CK-0%K groups was significantly higher than all other CK groups and their corresponding PI groups (*p* < 0.05). For PI plants, the leaf T-AOC in PI-50%P, PI- 25%P, and PI-0%P was significantly lower than in their corresponding CK groups (*p* < 0.05). The root T-AOC in PI-50%P was significantly higher than in CK-50%P, and the root T-AOC in CK-0%P was significantly higher than in PI-0%P (*p* < 0.05). The root and leaf T-AOC in PI-50%P was significantly higher than in PI-100%P (*p* < 0.05). The leaf T-AOC in PI-25%P and PI-0%P was significantly higher than in PI-100%P, while the root T-AOC in PI-25%P was significantly lower than in PI-100%P (*p* < 0.05). The leaf T-AOC in PI-50%K was significantly higher than in CK-50%K (*p* < 0.05). However, the leaf T-AOC in PI-25%K and PI-0%K was significantly lower than in their corresponding CK groups (*p* < 0.05).

### 2.6. Effects of P. indica on the Anthocyanins and Flavonoids Contents in Banana Roots and Leaves Under Varying P and K Concentrations

**Figure 5:**
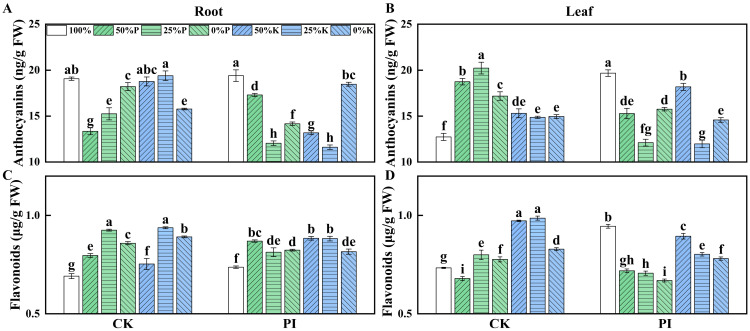
Effects of *P. indica* colonization on the anthocyanins (**A**,**B**) and flavonoid (**C**,**D**) contents in roots and leaves of banana plants under different concentrations of P and K treatments. CK: noncolonized control plants; PI: *P. indica*-colonized plants; FW: fresh weight. Different lowercase letters above columns represent significant differences among samples at *p* < 0.05 level.

Bioactive compounds, such as anthocyanins and flavonoids, play crucial roles in plant growth and development and stress responses. We compared the anthocyanins and flavonoids contents in roots and leaves of CK and PI banana plants under different concentrations of P or K treatments. For CK plants, the root anthocyanins content showed a ‘fall–rise’ trend with a decreasing P concentration, and a ‘fall–rise–fall’ trend with a decreasing K concentration (Figure 5A). Conversely, their leaf anthocyanin contents displayed the opposite change pattern (Figure 5B). For PI plants, both root and leaf anthocyanin contents exhibited a ‘fall–rise’ trend with a decreasing P or K concentration.

Root flavonoid contents in both CK-100% and PI-100% groups were significantly lower than in the other groups (*p* < 0.05) (Figure 5C). Leaf flavonoid content in CK-100% was significantly higher than in the CK-50%P, but significantly lower than in CK-25% and CK-0% (*p* < 0.05). However, the leaf flavonoid content in PI-100% was significantly higher than in all the other PI groups (*p* < 0.05). Except for the PI-100% and PI-50%P groups, which had significantly higher leaf flavonoid contents than their corresponding CK groups (*p* < 0.05), the leaf flavonoid contents in all the other PI groups were significantly lower than in the corresponding CK groups (*p* < 0.05) (Figure 5D).

### 2.7. Correlation Analysis Results of Banana Growth-Related and Physio-Biochemical Parameters

**Figure 6:**
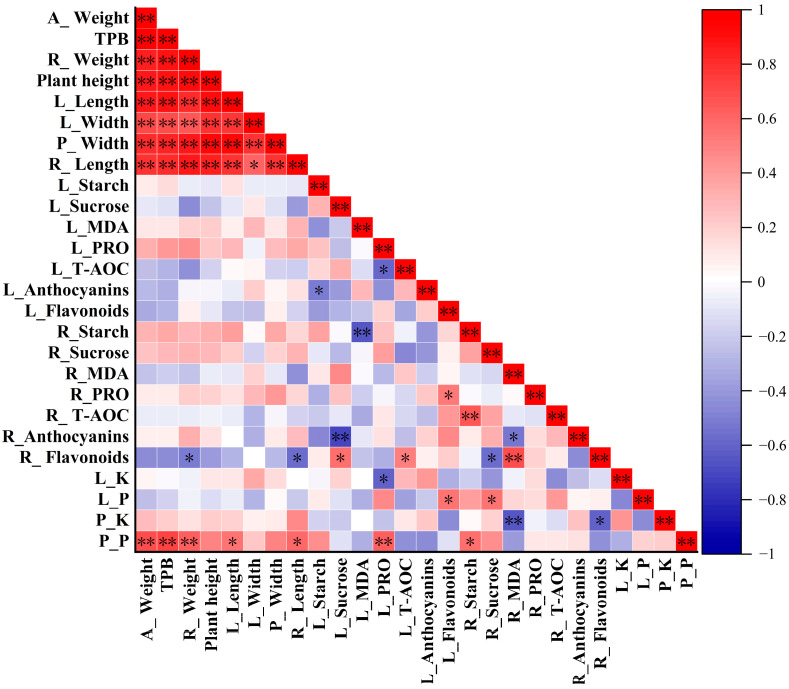
Correlation analysis results of banana growth-related and physio-chemical parameters under different concentrations of P and K treatments. A_: aboveground part; L_: leaf; P_: pseudo-stem; P: phosphorus; K: potassium; TPB: total plant biomass; MDA: malondialdehyde; PRO: proline; T-AOC: total antioxidant capacity. * and ** indicates significant (*p* < 0.05) and very significant (*p* < 0.01) correlation, respectively.

Correlation analysis was conducted to explore the complex relationships among banana growth parameters, root and leaf physio-biochemical parameters, and leaf and pseudo-stem P and K contents under varying P and K concentrations (Figure 6). Correlation analysis of the growth-related parameters revealed that, except for the root length and leaf width being significantly positively correlated (*p* < 0.05), all the other growth-related parameters showed very significant positive correlations (*p* < 0.01).

The pseudo-stem P content was very significantly positively correlated with aboveground part fresh weight, total plant biomass, root fresh weight, and leaf PRO content (*p* < 0.01), and significantly positively correlated with leaf length, root length, and root starch content (*p* < 0.05). The pseudo-stem K content was very significantly (*p* < 0.01) and significantly (*p* < 0.05) negatively correlated with root MDA content and root flavonoid content, respectively. Leaf P content was significantly positively correlated with leaf flavonoid content and root sucrose content (*p* < 0.05). The leaf K content was significantly positively correlated with the leaf PRO content (*p* < 0.05). These suggest that P and K play important roles in banana growth, carbohydrate storage, and secondary metabolism, and in osmoregulation under varying P and K concentrations.

The root flavonoids content was very significantly positively correlated with root MDA content (*p* < 0.01), significantly positively correlated with leaf sucrose content and leaf T-AOC (*p* < 0.05), but significantly negatively correlated with root fresh weight, root length, and root sucrose content (*p* < 0.05). Moreover, very significant negative correlations (*p* < 0.01) were identified between root anthocyanins content and leaf sucrose content, between root starch content and leaf MDA content; a very significant positive correlation (*p* < 0.01) was identified between root T-AOC and root starch content; significant negative correlations (*p* < 0.05) were identified between leaf T-AOC and leaf PRO content, between leaf anthocyanins content and leaf starch content, and between root anthocyanin content and root MDA content; and a significant positive correlation (*p* < 0.05) was identified between root PRO content and leaf flavonoids content. These findings indicated that the secondary metabolites and osmoprotectants accumulation dynamics contributed to balancing stress adaptation and growth under nutrient deficiency conditions.

Although no significant correlation was identified, root starch, sucrose, PRO, and anthocyanins contents, along with leaf PRO and MDA contents and pseudo-stem K content, were found to be positively correlated with aboveground part fresh weight, root fresh weight, and total plant biomass. Conversely, leaf sucrose, anthocyanins, and P contents; leaf and root T-AOC; and root MDA content exhibited negative correlations with plant biomass-related parameters. Additionally, root starch content was positively correlated with leaf starch content.

### 2.8. Principal Component Analysis (PCA) Results

**Figure 7:**
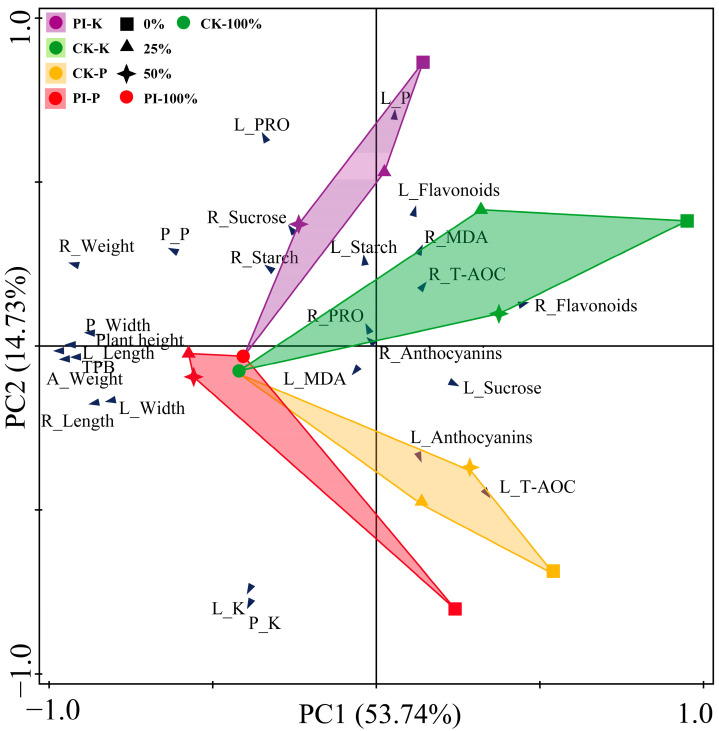
PCA results of banana growth-related and physio-chemical parameters under different concentrations of P and K treatments. P: phosphorus; PCA: principal component analysis; A_: aboveground part; L_: leaf; P_: pseudo-stem; P: phosphorus; K: potassium; TPB: total plant biomass; MDA: malondialdehyde; PRO: proline; T-AOC: total antioxidant capacity. CK: noncolonized control plants; PI: *P. indica*-colonized plants.

PCA was performed using the growth-related and physio-biochemical parameters of CK and PI banana plants treated with varying P or K concentrations (Figure 7). A clear separation among CK-P, CK-K, PI-P, and PI-K groups across different P or K concentrations was observed, underscoring the substantial influence of P, K, and *P. indica* colonization on banana growth-related and physio-biochemical parameters. In CK plants, growth-related parameters exhibited the strongest positive correlation with the CK-100% group, but negative correlations with other groups. Conversely, in PI plants, these parameters were positively correlated with PI-P and PI-K groups (excluding PI-0%P and PI-0%K groups). These suggested that *P. indica* colonization promoted banana growth across different P or K concentrations.

The first two principal components (PC1 and PC2) explained, respectively, 53.74% and 14.73% of the variances, cumulatively accounting for 68.47% of the total variance. PC1 encompassed all the growth-related parameters, as well as the pseudo-stem P content, root flavonoid content, and leaf sucrose content. All these parameters, except root flavonoids content and leaf sucrose content, showed positive correlations with the PI-P groups (excluding PI-0%P), CK-100% group, and PI-50%K group. Moreover, except for root length and leaf width, all the growth-related parameters also showed positive correlations with the PI-25%K group. Root flavonoid content showed positive correlations with PI-0%K and PI-25%K groups, while leaf sucrose content showed a positive correlation with the PI-0%P group. PC2 comprised all other physio-biochemical parameters. Parameters such as root and leaf starch and PRO contents, and root sucrose and anthocyanins contents were positively correlated with PI-P groups (excluding PI-0%P) and PI-K groups, but negatively correlated with CK-P groups and the PI-0%P group. The leaf P content, leaf and root flavonoid contents, root MDA content, and T-AOC showed positive correlation with CK-K groups (excluding CK-100%) and PI-K groups (excluding PI-100%). The leaf T-AOC, leaf anthocyanins, and sucrose contents were positively correlated with the CK-P groups (excluding CK-100%) and PI-0%P group. The leaf and pseudo-stem K contents and leaf MDA content were positively correlated with PI-P groups (excluding PI-100%).

### 2.9. Redundancy Analysis (RDA) Results

**Figure 8:**
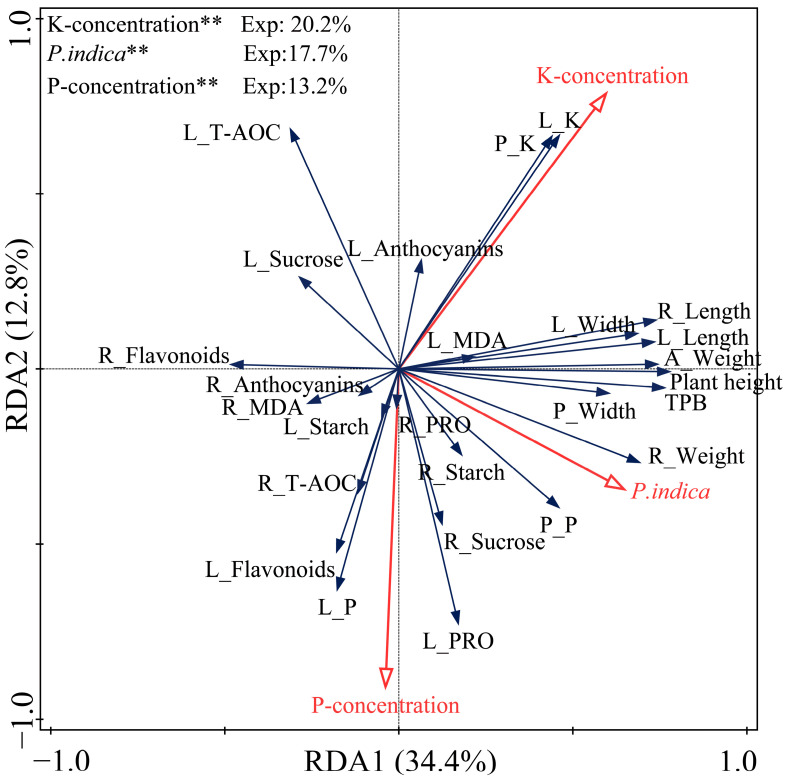
RDA results of banana growth-related and physio-chemical parameters under different concentrations of P and K treatments. RDA: redundancy analysis; P: phosphorus; K: potassium; A_: aboveground part; L_: leaf; P_: pseudo-stem; TPB: total plant biomass; MDA: malondialdehyde; PRO: proline; T-AOC: total antioxidant capacity. ** represents statistically significant factor influencing the variations in growth-related and physio-biochemical parameters of banana plants.

RDA of banana growth-related and physio-biochemical parameters was also conducted (Figure 8). RDA1 and RDA2 explained 34.4% and 12.8% of the variations, respectively. The K concentration was the most substantial contributor to the variations in growth-related and physio-biochemical parameters, explaining 20.2% of the total variation, followed by *P. indica* colonization (17.7%) and P concentration (13.2%). Both K concentration and *P. indica* colonization showed significant positive correlations with all growth-related parameters. The P concentration exhibited a significant positive correlation with all growth-related parameters except root length, leaf length, and leaf width. T-AOC and leaf sucrose and anthocyanins contents were significantly positively correlated with K concentration but negatively correlated with P concentration and *P. indica* colonization. Leaf and pseudo-stem K contents and leaf MDA content were significantly positively correlated with K concentration and *P. indica* colonization, but negatively correlated with P concentration. Leaf and root PRO contents, root starch and sucrose contents, root T-AOC, leaf and pseudo-stem P contents, and leaf flavonoids and starch contents were significantly positively correlated with P concentration and *P. indica* colonization. Moreover, root flavonoids and starch contents, and root MDA content were positively correlated with P concentration but negatively correlated with K concentration and *P. indica* colonization.

## 3. Discussion

### 3.1. P. indica Promoted the Growth of Banana Plants Under Different Concentrations of P and K Treatments

Banana plants are highly sensitive to an insufficient P or K supply [15,26]. Under such conditions, their growth and biomass accumulation are greatly inhibited [25,28,29]. In this study, both clean river sand-cultivated CK and PI banana plants, especially the CK plants, showed necrosis and yellowing or browning symptoms in old leaves with decreased P or K concentration. This finding highlighted the crucial roles of P and K in the plant growth of banana. Moreover, the root systems of *P. indica*-colonized banana plants were found to be larger than their corresponding noncolonized controls under different concentrations of P or K treatments, indicating that the fungal colonization improved the root development of banana plants under different nutrient conditions.

Inoculating endophytic fungi can enhance the plant height, root number, and dry matter accumulation [30]. *P. indica* has demonstrated the ability to improve P nutrition in host plants under P-limited conditions [9,31]. *P. indica*-colonized *Camellia oleifera* plants showed enhanced P uptake, higher expression of phosphate transporter genes, and improved soil phosphatase activities [32]. The aboveground part fresh weight of *P. indica*-colonized lettuce under 50%P treatment was comparable to that of lettuce without *P. indica* colonization grown under 100%P treatment [33]. Additionally, *P. indica* can improve plant growth and stress resistance by enhancing the absorption of K, by mediating the sodium (Na^+^) and potassium ion (K^+^) balance, and by influencing transcription of K transportation and absorption-related genes [34,35,36]. In our study, compared to CK-100%, the root length, root number, and pseudo-stem width of banana plants of PI-100% significantly increased. Notably, the root length, root number, and root fresh weight of the PI-100% group were the highest among all the groups, indicating that, at 100%P, *P. indica* colonization significantly enhanced the root development of banana plants. Furthermore, most growth-related parameters of *P. indica*-colonized banana plants were superior to those of uncolonized controls under the same P or K concentration, suggesting that the fungal colonization promotes banana growth under various P and K conditions. Under 0%P and 0%K conditions, all growth-related parameters of banana plants in both CK and PI groups were significantly reduced, highlighting the critical role of P and K in banana growth. Moreover, the growth-related parameters of PI-0%P and PI-0%K groups were higher than their corresponding CK groups, indicating that *P. indica* colonization improved the tolerance of banana to P and K deficiency.

It is worth noting that our PCA results revealed that all the growth-related parameters were positively correlated with PI-100%, PI-50%P, PI-25%P, PI-50%K, and PI-25%K groups. Consistently, our RDA results showed that *P. indica* colonization was significantly positively correlated with all growth-related parameters. These findings indicated again that the fungal colonization is helpful for the growth of banana plants under different concentrations of P and K treatments.

### 3.2. P. indica Colonization Influences Starch and Sucrose Accumulation in Banana Leaves and Roots Across Different P and K Concentrations

Carbohydrates are crucial for supporting plant biological processes and reflecting their environmental adaptability [37]. P is essential for photosynthesis, thereby greatly influencing the biosynthesis and accumulation of sucrose and starch in different plant organs. Previous studies showed that P deficiency led to carbohydrate accumulation in *Arabidopsis thaliana* leaves and excessive starch accumulation in rice stems [38]. K is required for activating enzymes involved in photosynthesis, carbohydrate biosynthesis, etc. [14], and it positively contributes to plant sugar metabolism [39]. Under K deficiency conditions, the sugar export in plant leaves was reconfigured [40]. In *Ocimum tenuiflorum*, AMF treatment significantly altered carbon compound accumulations [41]. In this study, the starch contents in banana leaf and root showed a positive correlation across different P and K concentrations, whereas the sucrose contents in leaf and root were negatively correlated. The root sucrose content was positively correlated, while the leaf sucrose content was negatively correlated with most growth-related parameters. The root starch content was also positively correlated to root and aboveground part fresh weights, and some other growth-related parameters. However, leaf starch content was negatively correlated with some growth parameters. These findings indicate that appropriate sucrose and starch allocations are crucial for banana growth. Notably, as P concentrations decreased, the starch content in CK roots and leaves followed a ‘fall–rise’ pattern, while an opposite starch content change pattern was observed in the PI groups. This suggests that *P. indica* colonization alters the starch accumulation changes in banana leaves and roots caused by a decrease in P concentration.

### 3.3. P. indica Colonization Influenced the Accumulations of Osmoprotectants and Secondary Metabolites in Banana Under Low and Deficient P and K Conditions

The accumulations of osmoprotectants and antioxidant capacity increased greatly under stress conditions to scavenge ROS and mitigate cell membrane damage [42,43,44]. Endophytic fungi colonization can reduce hydrogen peroxide (H_2_O_2_) levels, activate antioxidant metabolism, and improve osmoprotectants accumulation, thereby alleviating stress-induced damages and strengthening host plant stress tolerance [30,34]. In this study, root and leaf MDA and PRO contents were found to be positively correlated with most growth-related parameters, indicating that *P. indica* promotes banana plant growth by regulating MDA and PRO accumulations.

Evidence revealed that AMF can promote the accumulation of plant secondary metabolites, thereby improving host plant stress tolerance [41,45]. As an AMF-like fungus, *P. indica* also significantly influences host plants’ secondary metabolism [46,47]. *P. indica*-colonized *Pinus taeda* seedlings accumulated higher levels of some flavonoids and organic acids under drought stress [46]. In soybean, *P. indica* inoculation resulted in significant changes in root phenylpropanoids metabolism and in the expression of secondary metabolism-related genes [47]. In the fruit peel of *P. indica*-colonized passion fruit, the content of stress-resistant secondary metabolites significantly increased [48]. *P. indica* induced metabolic reprogramming in rice, greatly improving host plant tolerance to arsenic stress [49]. Flavonoids and anthocyanins play vital roles in plant adaptation and resistance to the external environment [50]. Our correlation analysis revealed that the root flavonoid content and leaf anthocyanin content were negatively correlated with biomass-related parameters. At 25%P, 0%P, 25%K, and 0%K, the root and leaf flavonoid contents in PI groups were significantly lower than in the corresponding CK groups. Moreover, leaf anthocyanin contents in PI-50%P, PI-25%P, PI-0%P, and PI-25%K groups were significantly lower than in the corresponding controls. The significant changes in flavonoids and anthocyanins between CK and PI plants across different P or K concentrations (particularly under 25% and 0% P or K conditions) suggest that *P. indica* colonization rebalances the secondary metabolites accumulation and promotes plant growth under nutrient stress.

## 4. Materials and Methods

### 4.1. Plant and Fungal Materials

The tissue-cultured ‘Baxijiao’ banana (*Musa acuminata* cv. ‘Baxijiao’) seedlings and the *P. indica* strain used in this study were provided by the Institute of Horticultural Plant Biotechnology, Fujian Agriculture and Forestry University. According to the method of Cheng et al. [22], the *P. indica* inoculation solution was prepared. Briefly, *P. indica* was inoculated onto potato dextrose agar (PDA) plates and incubated in the dark at 28 °C for 7 d. Then, three *P. indica* plugs (diameter = 5 mm) were taken from the edge of colonies, added to potato dextrose broth (PDB), shake-cultured at 200 rpm for 3 d, and diluted three times to obtain the inoculation solution.

### 4.2. P. indica Inoculation

In November 2019, after hardening, unique tissue-cultured banana seedlings were categorized into PI and CK groups, with each group containing approximately 300 banana seedlings. The roots of the PI seedlings were immersed in the *P. indica* inoculation solution for 6 h, while the roots of the CK group were immersed in an equally diluted PDB solution. Subsequently, banana seedlings were transplanted into pots (14 cm in diameter and 11 cm in height) containing clean river sand and cultured in a growth chamber at 25 °C, with a humidity of 60–70% and a light intensity of 160 mol·m^2^·s^−1^. Seedlings were watered with 50 mL of Hoagland solution twice a week. Two weeks post-*P. indica* inoculation, the trypan blue staining method was used to detect *P. indica* colonization in roots of banana seedlings from the PI groups [51]. Only the PI seedlings successfully colonized by *P. indica* were used for further experiments.

### 4.3. Plant Treatments with Different Concentrations of P and K

Banana seedlings at four leaves and one emerging leaf stage were subjected to phosphorus treatments by watering them twice a week using Hoagland solution with different concentrations of P and K (pH = 6.0) [52,53]. To maintain the nitrogen (N) content in K-deficient nutrient solutions, an appropriate amount of sodium nitrate (NaNO_3_) solution was added [52,53]. Four P and K concentration gradients (100%, 50%, 25%, and 0%) were designed, with at least twenty-four CK and PI banana plants for each gradient.

### 4.4. Measurements of Growth-Related Parameters

Six months post-P or K treatments, growth-related parameters, including plant height (the distance from the pseudo-stem base to the top), leaf length and width, root length (the longest length of roots), root fresh weight, aboveground part fresh weight, and total plant biomass of banana plants from different groups were measured. The plant height, leaf length and width, and root length were measured using a ruler, and the weights of root, aboveground part, and whole plant were measured with an electronic balance (HAT-A+100, Huazhi (Fujian) Electronics Technology Co., Ltd., Putian, China). For each parameter, at least eight banana plants from each group were measured.

### 4.5. Determinations of P and K Contents in Banana Leaf and Pseudo-Stem

Banana leaves and pseudo-stem samples were cleaned, dried to constant weight, ground into fine powders, and subjected to determinations of P and K using the inductively coupled plasma–mass spectrometry method [54]. For each group, three biological replicates were determined, and each replicate is a mixture of three banana plants.

### 4.6. Determinations of Starch and Sucrose Contents

P and K limitation or deficiency will influence the carbohydrate metabolism and transportation of plant species. To investigate the influences of *P. indica* colonization on the carbohydrate metabolism in banana plants under different concentrations of P and K treatments, the starch and sucrose contents in leaves and roots of banana plants from different groups were determined using colorimetry methods using a spectrophotometer (UV-1800, Max-analytical Instruments, Shanghai, China) [55]. For each group, three replications were made, each of which consisted of a mixture of three banana plants.

### 4.7. Determinations of Malondiadehyde (MDA), Proline (PRO), Anthocyanin, and Flavonoid Contents and Total Antioxidant Capacity (T-AOC)

Osmoprotectants and bioactive compounds contributed a lot to the plant responses to low nutrient and nutrient deficiency stresses. Therefore, in this study, we determined the MDA, PRO, anthocyanin, and flavonoid contents to explore their accumulation changes in roots and leaves of both CK and PI banana plants under different concentrations of P and K treatments. The malondiadehyde (MDA) contents in banana roots and leaves were determined by using the thiobarbituric acid (TBA) method [56]. The PRO content determination was conducted according to Bate et al. [57]. By using commercial kits produced by Suzhou Keming Biotechnology Company (Suzhou, China), the anthocyanins and flavonoids contents were determined using a spectrophotometer (UV-1800, Max-analytical Instruments, Shanghai, China).

Fungal colonization and abiotic stresses will greatly influence the ROS scavenging ability of plant species. In this study, the T-AOC in roots and leaves of banana plants from different groups was also determined using the ferric-reducing antioxidant power (FRAP) method. For each parameter, three biological replicates were determined, with each replicate being a mixture of three banana plants.

### 4.8. Statistical Analyses

By using Excel and SPSS 27.0 (IBM, Armonk, NY, USA), the data obtained in this study were analyzed. All the results were represented as mean ± standard deviation of at least three replicates. By using Duncan’s method of SPSS 27.0, the differences in significance of the growth-related and physio-biochemical parameters among banana plants from different groups were analyzed. PCA and RDA of these parameters were conducted by using OriginPro 2021 (Northampton, MA, USA) and Canoco (Version 5.0, Microcomputer Power Corporation, Ithaca, NY, USA) [58], respectively.

## 5. Conclusions

This study demonstrated that *P. indica* can promote the growth of banana plants under different concentrations of P and K treatments by mediating the starch and sucrose accumulation in roots and leaves, and by modulating the accumulation of stress markers (such as MDA, anthocyanins, etc.). Our study will be helpful for understanding the plant-growth-promoting mechanisms of *P. indica* in banana under both normal and P or K-limited conditions and can provide a basis for the applications of *P. indica* in banana cultivation practices.

## Figures and Tables

**Table 1:** Effects of *P. indica* on the growth of banana under varying P and K concentrations. Within the same column, different lowercase letters indicate significant difference (*p* < 0.05) among different groups.

Groups	Plant Height (cm)	Leaf Length (cm)	Leaf Width (cm)	Pseudo-Stem Width (cm)	Root Length (cm)	Root Fresh Weight (g)	Aboveground Part Fresh Weight (g)	Total Plant Biomass (g)
CK-100%	19.15 ± 1.02 b	23.96 ± 1.09 bc	9.25 ± 0.90 cd	1.31 ± 0.05 abcd	45.85 ± 8.84 a	14.48 ± 1.23 abc	53.07 ± 8.50 b	67.55 ± 9.78 ab
PI-100%	20.67 ± 1.55 b	23.83 ± 1.55 bc	9.78 ± 0.60 bc	1.48 ± 0.28 ab	46.20 ± 6.25 a	18.30 ± 5.23 a	49.04 ± 3.05 bc	67.33 ± 5.49 ab
CK-50%P	15.78 ± 1.21 de	21.23 ± 1.41 de	9.28 ± 0.66 cd	1.23 ± 0.10 cd	38.60 ± 1.54 abc	7.55 ± 2.19 def	31.81 ± 6.96 ef	39.37 ± 7.31 de
PI-50%P	23.43 ± 1.22 a	25.87 ± 1.91 b	10.71 ± 0.98 ab	1.44 ± 0.04 abc	43.38 ± 7.41 ab	17.10 ± 3.48 ab	65.98 ± 7.15 a	83.07 ± 10.2 a
CK-25%P	17.14 ± 0.80 cd	22.98 ± 1.55 cd	9.92 ± 1.35 bc	1.31 ± 0.11 abcd	38.12 ± 8.66 abc	9.71 ± 2.79 cdef	35.66 ± 8.40 cdef	45.38 ± 8.45 cd
PI-25%P	24.52 ± 1.57 a	28.73 ± 2.62 a	11.49 ± 1.01 a	1.54 ± 0.14 a	43.13 ± 2.73 ab	16.39 ± 5.32 ab	67.29 ± 8.90 a	83.68 ± 14.21 a
CK-0%P	12.41 ± 1.99 fg	18.12 ± 1.31 fg	6.81 ± 1.17 fg	1.10 ± 0.14 de	33.10 ± 1.50 cde	6.08 ± 0.86 ef	26.42 ± 9.84 ef	32.50 ± 8.98 de
PI-0%P	13.81 ± 1.54 ef	20.40 ± 1.99 e	9.72 ± 1.00 bc	1.12 ± 0.25 de	32.77 ± 1.05 cde	6.80 ± 0.94 def	39.09 ± 5.35 cde	45.89 ± 6.21 cd
CK-50%K	14.69 ± 1.78 e	19.18 ± 1.48 ef	7.72 ± 0.56 ef	1.17 ± 0.14 de	26.67 ± 2.31 e	9.29 ± 2.41 cdef	27.16 ± 8.69 ef	36.45 ± 10.73 de
PI-50%K	18.92 ± 1.57 bc	24.76 ± 1.69 bc	9.98 ± 0.68 bc	1.52 ± 0.08 a	40.27 ± 3.90 abc	13.92 ± 2.20 abc	46.57 ± 10.50 bcd	60.49 ± 12.62 bc
CK-25%K	15.65 ± 1.46 de	21.01 ± 1.21 de	8.27 ± 0.75 de	1.26 ± 0.35 bcd	27.35 ± 4.47 de	9.25 ± 3.07 cdef	33.19 ± 9.14 def	42.44 ± 12.04 de
PI-25%K	16.71 ± 1.51 d	20.57 ± 1.68 e	9.52 ± 0.95 bc	1.25 ± 0.16 bcd	36.27 ± 2.23 bcd	11.69 ± 3.96 bcde	36.77 ± 10.86 cde	48.47 ± 14.82 cd
CK-0%K	11.30 ± 1.62 g	16.70 ± 2.21 g	6.56 ± 0.85 g	0.95 ± 0.10 e	27.03 ± 2.89 e	5.20 ± 1.73 f	22.23 ± 2.62 f	27.43 ± 4.27 e
PI-0%K	14.16 ± 1.23 ef	20.79 ± 2.02 de	7.88 ± 0.53 ef	1.16 ± 0.15 de	34.65 ± 4.39 bcde	12.10 ± 4.81 bcd	36.15 ± 9.22 cdef	48.26 ± 11.69 cd

## Data Availability

The data supporting the reported results can be found in the manuscript.

## Abbreviations

The following abbreviations are used in this manuscript:

CK	noncolonized control
DW	dry weight
FW	fresh weight
K	potassium
MDA	malondialdehyde
P	phosphorus
PCA	principal component analysis
PDA	potato dextrose agar
PDB	potato dextrose broth
PI	*Piriformospora indica*-colonized
PRO	proline
RDA	redundancy analysis
ROS	reactive oxygen species
T-AOC	total antioxidant capacity
TPB	total plant biomass
